# Maternal age and density shape offspring foraging strategies in a predatory mite

**DOI:** 10.1093/beheco/arag018

**Published:** 2026-02-17

**Authors:** Keshi Zhang, Peter Schausberger, Zhi-Qiang Zhang

**Affiliations:** School of Biological Sciences, University of Auckland, 3A Symonds Street, Auckland 1010, New Zealand; Bioeconomy Science Institute, Manaaki Whenua – Landcare Research Group, 231 Morrin Road, Auckland 1072, New Zealand; Department of Behavioral and Cognitive Biology, University of Vienna, 1 Djerassiplatz, Vienna 1030, Austria; School of Biological Sciences, University of Auckland, 3A Symonds Street, Auckland 1010, New Zealand; Bioeconomy Science Institute, Manaaki Whenua – Landcare Research Group, 231 Morrin Road, Auckland 1072, New Zealand

**Keywords:** *Amblyseius herbicolus*, boldness, maternal effects, personality, predation behavior, prey consumption, transgenerational plasticity

## Abstract

Maternal effects are key drivers of offspring phenotypic plasticity, influencing traits such as survival, growth, and behavior. Maternal age at oviposition is an intrinsic factor governing such effects, which often exerts negative impacts on offspring traits. However, in the thelytokous predatory mite *Amblyseius herbicolus*, offspring of older mothers exhibit increased growth efficiency and reduced prey consumption. The proximate mechanisms of this inverse Lansing effect remain elusive, but this conservative offspring’ foraging strategy may reflect an anticipatory maternal response to mitigate intraspecific competition among later-produced offspring. Here, we investigated how maternal age at oviposition and maternal rearing density influence offspring foraging strategies. Eggs (ie offspring) were collected from mothers maintained under low- and high-density conditions and classified as Young or Old based on maternal age at oviposition. Offspring were then assessed for immature survival under low prey availability, prey consumption, predation incidence, latency to attack prey, and superfluous killing. Offspring of older mothers showed reduced prey consumption and lower predation incidence, whereas offspring of high-density mothers had higher survival under prey limitation. Maternal density partially modulated age-related effects. Our findings highlight the role of maternal effects in shaping adaptive foraging strategies and demonstrate that maternal influences can induce risk-averse behavioral changes in response to ecological conditions. Both intrinsic and extrinsic maternal factors shape offspring behavioral strategies, and maternal age, in particular, can serve as a dynamic source of variation influencing predator–prey interactions and population dynamics.

## Teaser

Parents' experiences shape how their offspring behave. In a laboratory study, we found that mother mites' age and crowded living conditions influenced how their young hunted and ate prey. Offspring of older or crowded mothers were more cautious hunters. These results show that a mother's experiences can directly shape her offspring's behaviour, potentially helping them cope with changing environments.

## Introduction

Organisms cope with environmental variability through phenotypic plasticity, the ability of a single genotype to express different phenotypes in response to changing conditions (West-Eberhard 2003). Beyond an individual's own plastic responses, maternal effects allow mothers to influence offspring phenotypes through diverse, mutually nonexclusive, proximate mechanisms such as differential provisioning, hormonal signaling, or epigenetic regulation, with important consequences for offspring size, development, stress tolerance, survival, and behavior (Bonduriansky and Day 2009; Bonduriansky 2012; Bell and Hellmann 2019).

Among maternal factors, maternal age is a well-recognized determinant of offspring performance (Plaistow et al. 2015; Perez et al. 2017; Hernández et al. 2020). Offspring quality often declines with maternal age, a phenomenon known as the Lansing effect, which can manifest as reduced hatching success, smaller size, lower stress resistance, or shortened lifespan (Lansing 1947; Fox et al. 2003; Schroeder et al. 2015; Moorad and Nussey 2016; Krug et al. 2020; Reichert et al. 2020; Ivimey-Cook et al. 2023). Lansing effects may arise from maternal senescence or age-related changes in reproductive strategy (Goos et al. 2019; Monaghan and Metcalfe 2019). However, inverse Lansing effects—although rare—have also been reported, where offspring of older mothers show enhanced performance (eg spiders and beetles) (Ameri et al. 2019; Singh et al. 2021; Anderson et al. 2022). Such variation suggests that maternal age effects are species-specific and/or context-dependent (Plaistow and Benton 2009; van Daalen et al. 2022).

In the predatory mite *Amblyseius herbicolus* (Chant) (Acari: Phytoseiidae), an inverse Lansing effect has been observed: offspring of older mothers survived better, were larger at maturity, and consumed fewer prey (Zhang et al. 2025). These responses suggest that maternal age may mediate offspring foraging strategies, potentially as an adaptive response to changing environments. Advanced maternal age at oviposition could act as a predictive cue for deteriorating environments such as low prey density or high-density of conspecifics (Vargas et al. 2012; Rossi et al. 2016), which could favor the production of offspring with more conservative or risk-averse foraging behaviors.

Population density in the maternal environment is another key factor influencing offspring phenotypic traits (Dantzer et al. 2013; Bian et al. 2015). For example, red squirrel (*Tamiasciurus hudsonicus*) mothers exposed to high-density cues produced faster-growing offspring (Dantzer et al. 2013) and a similar pattern has been reported in parasitoid wasps (*Copidosoma koehleri*) (Morag et al. 2011). Higher population density can accelerate resource depletion and intensify intraspecific aggression such as cannibalism, which is common in *A. herbicolus* (Zhang and Zhang 2022a, 2022b; Zhang and Zhang 2023). If a conservative foraging phenotype is associated with post-hatching density, *A. herbicolus* mothers experiencing prolonged crowding may adjust offspring provisioning or development to prepare them for intensified intraspecific competition or limited resources, irrespective of maternal age. Such maternal adjustments could result in offspring that either require fewer prey items or consume prey more efficiently. Indeed, wasteful predation or superfluous killing—where prey are killed but only partially consumed—has been documented in mites and other predators (Metz et al. 1988; Riechert and Maupin 1998; Maupin and Riechert 2001; López-Mercadal et al. 2024).

Building on this framework, we tested how maternal age and rearing density influence the foraging strategies of *A. herbicolus* offspring. Specifically, we asked whether maternal conditions shape (i) offspring survival under limited prey supply, (ii) offspring prey consumption under ample prey availability, (iii) their predation propensity (likelihood and latency of attack), and (iv) the occurrence of superfluous killing.

We tested 4 hypotheses regarding maternal effects on offspring foraging behavior. (i) Offspring of older mothers would consume less prey under ample prey availability, reflecting a more conservative foraging strategy; correspondingly, they would show increased survival to adulthood under restricted prey availability. (ii) Predation propensity would be reduced in offspring of older mothers, consistent with a more risk-averse behavioral phenotype. (iii) Superfluous killing would be more frequent in offspring of younger mothers, reflecting a more wasteful foraging strategy. (iv) The influence of maternal age on offspring would weaken under high maternal density, where offspring of younger mothers would also adopt a more conservative foraging phenotype.

By examining how maternal age and maternally experienced population density shape offspring foraging strategies, this study provides insights into the ecological significance of maternal effects, emphasizing their role in shaping behavioral plasticity that may influence predator–prey interactions and population dynamics.

## Materials and methods

### Study animals and rearing conditions

**Figure 1:**
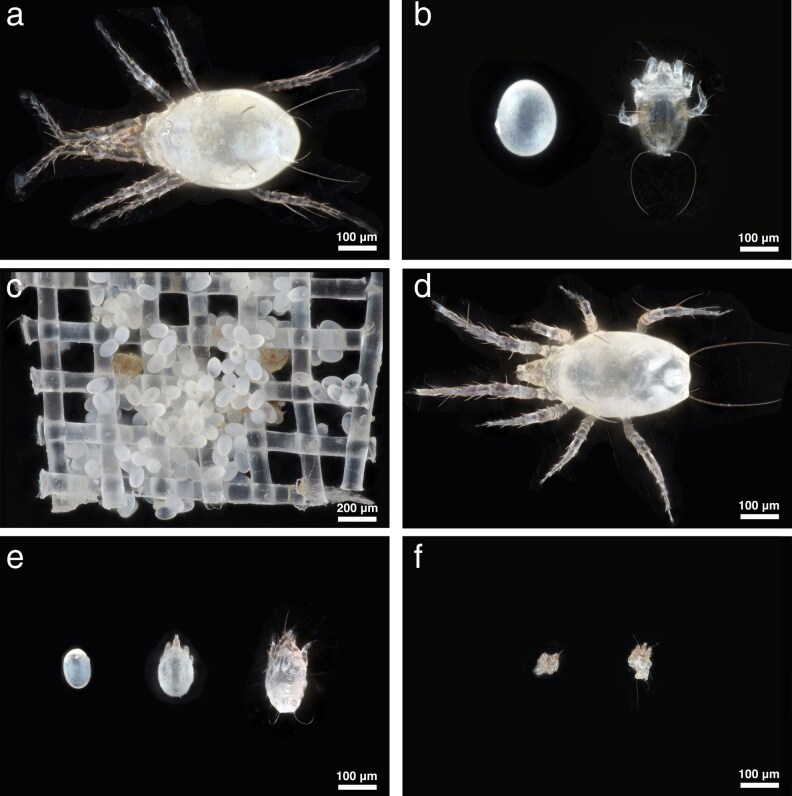
The test subjects used in this study. a) A gravid *Amblyseius herbicolus* female. b) From left to right: egg and larva of *A. herbicolus*. c) A mesh containing *Carpoglyphus lactis* eggs (mostly) and other life stages. d) A newly molted adult of *A. herbicolus*. e) From left to right: egg, larva, and protonymph of *C. lactis*. f) Shrank larvae of *C. lactis* after feeding by *A. herbicolus* larvae. Images captured with a 20× adapted lens for (a), (b), (d to f), and a 10× adapted lens for (c).

*Amblyseius herbicolus* is a generalist predatory mite (<500 μm in body length) (Fig. 1a) and develops through 5 life stages: egg, larva, protonymph, deutonymph, and adult (McMurtry et al. 2013; Zhang and Zhang 2021). Individuals of *A. herbicolus* could complete development within approximately 1 week and have a lifespan of around 1 month under laboratory conditions (Liu et al. 2024a). A laboratory population was established from >30 adult females collected on avocado (*Persea americana* Mill.) leaves in Te Puna, Tauranga, New Zealand. Species identity was confirmed morphologically (Ma et al. 2024). Colonies were maintained on an ad libitum supply of dried fruit mites, *Carpoglyphus lactis* (L.) (Acari: Carpoglyphidae) for about a year before conducting the experiment in May 2025.

*Carpoglyphus lactis* was obtained from Bioforce Ltd. (Auckland, New Zealand) and reared in bulk on a bran-based mixture of locally sourced ingredients consisting approximately 96% wheat bran (Edmonds, New Zealand), 1% icing sugar (Chelsea, New Zealand), and 3% dry yeast pellets (Edmonds, New Zealand). Individuals of *C. lactis* pass through 5 life stages: egg, larva, protonymph, tritonymph, and adult.

**Figure 2:**
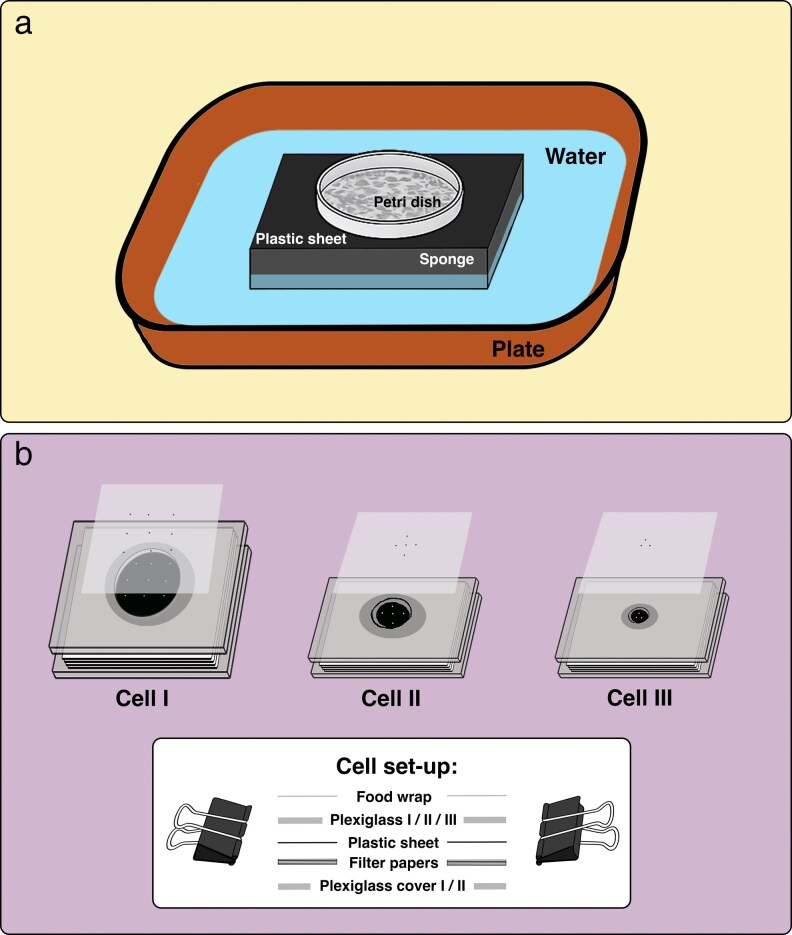
Experimental set-ups used in the study. a) Rearing set-ups for predator and prey stock cultures. b) Modified Munger cell set-ups used for experimental assays.

Predator and prey stock cultures were maintained in specially designed set-ups (Fig. 2a) under controlled conditions (25 °C ± 1 °C, 80% ± 5% RH, 16:8 h light–dark) (Zhang and Zhang 2021). The rearing system comprised a plastic container (225 mm × 225 mm × 35 mm; length × width × height) filled with water. A Petri dish (55 mm in diameter), the main housing arena, was placed on top of a black plastic sheet, which was positioned on a sponge (100 mm × 100 mm × 30 mm) and placed in the center of the water-filled container. The black plastic sheet was used to improve mite visibility and prevent water overflow. The water in the container acted both as a water reservoir and barrier preventing mite escape. Inside the Petri dish, a mixture of wheat bran, icing sugar, and dry yeast pellets was used to rear *C. lactis*. Black plastic sheet squares (c. 10 mm × 10 mm) and black sewing threads (c. 50 mm long) were placed atop the substrate and around the Petri dish to provide resting sites for *A. herbicolus*. The bran mixture, mixed-stage *C. lactis*, and water were replenished weekly.

Three sizes of modified Munger cells (I–III) were used in the experiments to accommodate different predator life stages and purposes, providing sufficient space for the test subjects without compromising observation or predator foraging efficiency (Fig. 2b). The largest cell (Cell I) consisted of 2 plexiglass slides (38 mm × 38 mm × 3 mm; length × width × thickness). The upper slide (Plexiglass I) contained a centrally located hole (15 mm in diameter; 530.14 mm^3^ in volume) that served as a chamber for the mites. The medium-sized cell (Cell II) consisted of 2 plexiglass slides (38 mm × 25 mm × 2 mm; length × width × thickness). The upper slide (Plexiglass II) contained a centrally located, truncated cone-shaped hole (top diameter = 10 mm; bottom diameter = 7 mm; volume = 114.67 mm^3^) that served as a chamber for the mites. The smallest cells (Cell III) had the same sized plexiglass slides as Cell II, except that the truncated cone-shaped holes were smaller (top diameter = 5 mm; bottom diameter = 3 mm; volume = 25.66 mm^3^). The openings (ie arena) were covered with a layer of food wrap on the upper side with small, pierced holes (using a size 000 insect pin) to facilitate air exchange. One the bottom, cells were made with plastic discs that were 20 mm in diameter for Cell I and 10 mm in diameter for Cell II and III. A stack of 4 filter paper placed beneath the plastic film served as a water reservoir and to maintain the freshness of the leaf discs. The cells were assembled by securing the 2 slides together with metal clips.

### Experimental preparation

Eggs of *A. herbicolus* were collected by placing short black sewing threads (c. 30 mm long) into the stock cultures overnight. Fifty freshly laid eggs (<16 h old) were transferred to a new culture supplied with ad libitum *C. lactis* to establish age-synchronized cohorts. From Day 11 onwards, new threads were introduced daily for egg collection over a 7-day period.

**Figure 3:**
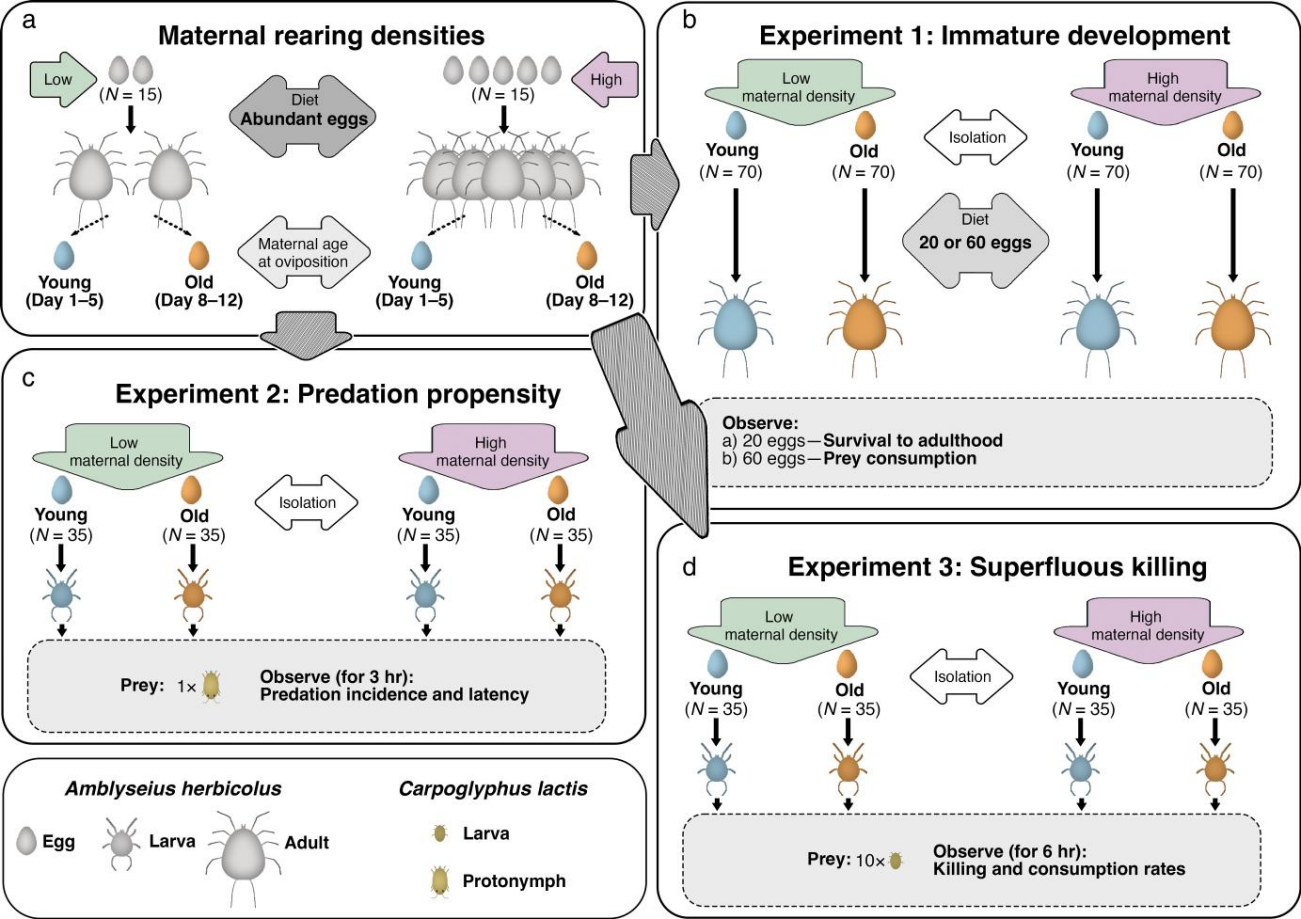
Schematic representation of the experimental design. a) Initial preparation of age-synchronized cohorts under different rearing densities. b) Experiments 1a and 1b: survival and prey consumption during immature development. c) Experiment 2: predation propensity. d) Experiment 3: superfluous killing.

To manipulate maternal population density, eggs of *A. herbicolus* were reared in Cell I at either Low (2 eggs) or High (5 eggs) population density, each with 4 threads (c. 10 mm long) per cell as refuge (Fig. 3a). Each density was replicated 15 times, with replacements added if mortality occurred before oviposition. After oviposition, all adult females survived until the end of the examination period (ie Day 12). Prey (*C. lactis*) were provided ad libitum as previously frozen mesh pieces containing >100 eggs and some mixed stages (Fig. 1c). The *C. lactis*-containing mesh was obtained using the method described in Liu et al. (2024b) and frozen at −18 °C for approximately 1 week. The mesh was then thawed at room temperature (c. 20 °C) for 10 min and cut into smaller pieces (c. 2 mm × 2 mm). One mesh piece per cell was supplied for Low-density and 2 for High-density cohorts, and the amount of mesh was doubled after individuals reached maturity (Fig. 1d). Meshes were replaced every 2 days; *A. herbicolus* found on the mesh were gently removed with a fine brush (size 000).

As *A. herbicolus* reproduces asexually (Zhang and Zhang 2022b, 2022c), females produce eggs without mating. Eggs laid were categorized as Young (Days 1 to 5 after the onset of oviposition) or Old (Days 8 to 12 after the onset of oviposition) based on maternal age at oviposition (Fig. 3a), with oviposition beginning approximately 10 days after egg hatch. Daily removal of eggs ensured a relatively stable maternal population density within each cell. The collected eggs were randomly allocated to egg size measurement or to Experiments 1 to 3. To maintain cell hygiene, adults were transferred into new cells 3 times: after reaching adulthood, after the first 5-day egg collection bout, and after the noncollection interval (ie Days 6 and 7).

### Egg size measurement

To assess maternal provisioning, 35 eggs per maternal age × population density treatment were randomly selected for size estimation. Egg dimensions were measured under a phase-contrast microscope (Eclipse 90i, Nikon Corporation, Japan) with NIS-Elements (version 5.10) at 200× magnification. The individual egg volume (*V*) was estimated using the length (*L*) and maximum breadth (*B*) of the eggs (Narushin 2005):


$$V=(0.6057\text{–}0.0018B)\times L\times B^{2}$$

### Experiment 1: offspring survival and prey consumption

Freshly laid eggs (<16 h old) from each maternal density treatment and maternal age were reared individually in Cell II with a short thread (c. 1 mm long) as refuge. A total of 70 predator eggs were used per treatment, with half assigned to Experiment 1a and half to Experiment 1b (Fig. 3b). Thawed *C. lactis* eggs (Fig. 1e) were provided daily as prey from the second day onwards. Individuals that failed to hatch (c. 6%) or were injured during molting (c. 1%) were excluded from the final analyses.

- Experiment 1a (survival): 20 prey eggs (4 per day for 5 days) were provided. Survival to adulthood was checked daily and recorded as a binary outcome (yes/no).
- Experiment 1b (consumption): 60 prey eggs (12 per day for 5 days) were provided. Cells were checked daily and total consumption during immature development was recorded.

Diet levels were based on preliminary trials showing approximately 20% survival to maturity with 20 eggs (representing extreme food limitation), and almost 100% survival with 60 eggs (representing abundant food supply) (Zhang and Zhang 2025; Zhang et al. 2025). For individuals given 20 eggs, a cluster of 4 eggs was placed in the center of the plastic sheet, whereas for individuals given 60 eggs, 3 clusters of 4 eggs each were placed around the central area of the plastic sheet.

### Experiment 2: predation propensity

To assess predation propensity, freshly laid eggs (<16 h old) from each maternal density treatment and maternal age were hatched individually in Cell III to obtain larvae (Figs. 1b and 3c). A total of 35 larval predators were used per treatment. Using larvae ensured standardized predator experience (ie prey-naïve and food-deprived). A mobile prey life stage was used to expose predators to prey movement and escape behavior, rather than immobile prey eggs. After a 15-min acclimation period, each larva was offered a single live *C. lactis* protonymph (Fig. 1e). Protonymphs were obtained by rearing *C. lactis* eggs in Cell I with abundant yeast. Prey presentation followed the same precautions as in Experiment 1.

Cells were checked every 15 min for 3 h. Predation incidence (yes/no) and latency to attack (time to first kill) were recorded.

### Experiment 3: superfluous killing

Freshly hatched larvae (<16 h old) were obtained using the same hatching procedure as in Experiment 2. A total of 35 larval predators were used per treatment. To assess superfluous killing, these larvae were individually placed in Cell III, acclimated for 15 min, and then offered 10 *C. lactis* larvae per cell (Figs. 1e and 3d). Larval prey were used to allow detection of superfluous killing, which cannot be reliably assessed using prey eggs and would be obscured by rapid satiation when using larger prey such as protonymphs. Prey larvae were prepared as in Experiment 2 but without yeast supplementation. Prey presentation followed the same precautions as in Experiment 1, with a cluster of 10 *C. lactis* larvae transferred at once to each cell using a fine brush to minimize disturbance. Partial consumption of prey items was determined by the partially deflated prey corpses and was used as indicator of superfluous killing.

Cells were checked every 30 min for 6 h, and the total prey killed and consumed partially or completely were recorded.

### General procedures

All brushes, pins, and cells were sterilized with 75% ethanol 24 h before use. Filtered water was added daily (Experiment 1) or at the start (Experiments 2 to 3) to maintain humidity of the cells. Handling was performed carefully to minimize disturbance; no flight response was observed except during brushing individuals off the meshes. For Experiments 2 and 3, observers were blind to treatments until after data collection (ie single-blind design).

### Statistical analysis

All analyses were performed in R (R Core Team 2024) using RStudio (version 2024.09.1). Figures were generated with *ggplot2* (Wickham 2016); estimated marginal means were calculated with *emmeans* (Lenth 2025). Egg size (volume) was analysed with Gaussian generalized linear models (GLMs). Survival (Experiment 1a) and predation incidence (Experiment 2) were analysed with binomial GLMs. Prey consumption (Experiments 1b, 3) was analysed with Poisson GLMs. Latency to attack (Experiment 2) was analysed with quasi-Poisson GLMs due to over dispersion. All models included maternal age and maternal density as fixed factors. Model assumptions (normality, dispersion, residual patterns) were checked. Pairwise comparisons were conducted using estimated marginal means. Egg sizes, prey consumption, and predation latencies were presented as medians with interquartile ranges (IQR). Statistical significance was set at *α* = 0.05.

## Results

### Egg size measurement

**Figure 4:**
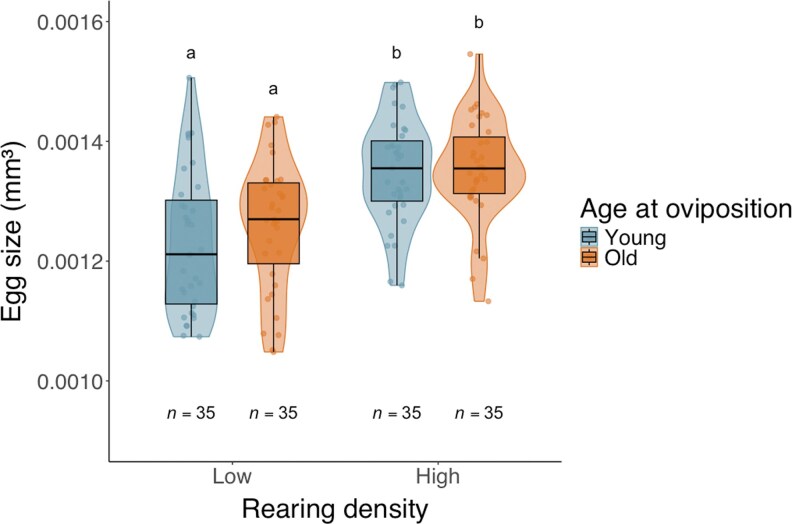
Egg size (measured in volume) of *Amblyseius herbicolus* under different rearing densities and at different ages during oviposition. Each violin shows the density of observations; dots represent individual eggs; boxplots indicate median, IQR, and 1.5 × IQR whiskers. Sample sizes (*n*) are indicated below each box. Different letters above the boxes denote significant differences from pairwise contrasts of estimated marginal means (*P* < 0.01).

The egg size was significantly influenced by maternal rearing density (GLMs: Wald χ^2^ = 15.270, df = 1, *P* < 0.001). Specifically, individuals reared under high densities produced larger eggs than those reared under low densities (Fig. 4). Maternal age at oviposition (Wald χ^2^ = 0.034, df = 1, *P* = 0.854) and the interaction between rearing density and age (Wald χ^2^ = 0.526, df = 1, *P* = 0.468) did not affect egg size.

### Experiment 1a: survival to adulthood

When given 20 prey eggs, offspring survival was significantly affected by maternal rearing density (GLMs: Wald χ^2^ = 5.512, df = 1, *P* = 0.017). Specifically, offspring from mothers reared under high density had a higher survival rate to adulthood than those from mothers reared under low density (Table 1). Maternal age at oviposition (Wald χ^2^ = 3.808, df = 1, *P* = 0.051) had a marginally significant effect on offspring survival, whereas the interaction between maternal rearing density and age was nonsignificant (Wald χ^2^ = 2.358, df = 1, *P* = 0.125).

**Table 1:** Survival to adulthood of *Amblyseius herbicolus* offspring whose mothers were reared under different densities and at different ages during oviposition.

Density	Age	*n*	Survival to adulthood (%)
**Low**	Young	33	72.7%^a^
	Old	33	90.9%^a,b^
**High**	Young	33	93.9%^b^
	Old	32	90.6%^a,b^

Each offspring was given 20 Carpoglyphus lactis eggs. Different letters (a, b) after percentage results denote significant differences from pairwise contrasts of estimated marginal means (*P* < 0.05).

### Experiment 1b: prey consumption

**Figure 5:**
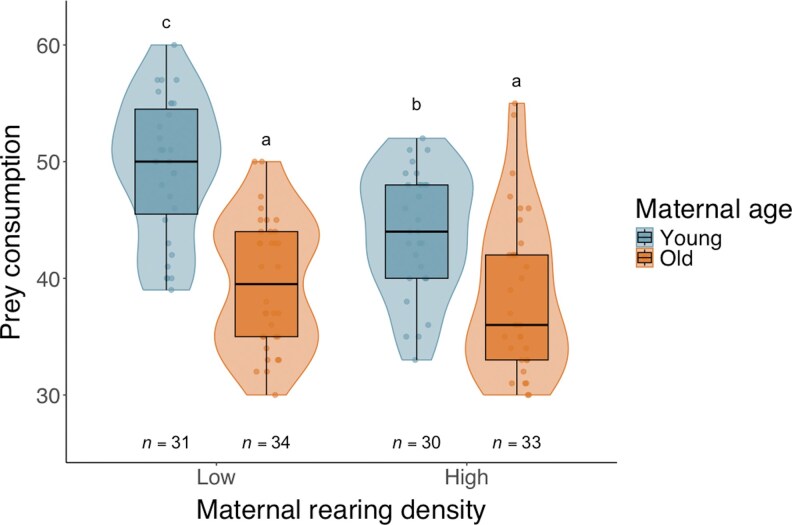
Prey consumption of *Amblyseius herbicolus* offspring whose mothers were reared under different densities and at different ages during oviposition. Each offspring was given 60 *Carpoglyphus lactis* eggs. Each violin shows the density of observations; dots represent individual consumptions; boxplots indicate median, IQR, and 1.5 × IQR whiskers. Sample sizes (*n*) are indicated below each box. Different letters above the boxes denote significant differences from pairwise contrasts of estimated marginal means (*P* < 0.05).

When given 60 eggs, offspring prey consumption was significantly influenced by maternal age at oviposition (GLMs: Wald χ^2^ = 10.568, df = 1, *P* = 0.001), where individuals from older mothers consumed fewer prey than those from younger mothers (Fig. 5). Maternal rearing density had no effect on offspring’ prey consumption (Wald χ^2^ = 0.388, df = 1, *P* = 0.533). The interaction between maternal rearing density and age at oviposition was marginally significant (Wald χ^2^ = 3.098, df = 1, *P* = 0.078). Among offspring of young mothers, those whose mothers were reared at high density exhibited significantly lower prey consumption than those from low-density-reared mothers (Fig. 5).

### Experiment 2: predation propensity

The offspring predation incidence towards a *C. lactis* protonymph during the larval stage was significantly affected by maternal rearing density (GLMs: Wald χ^2^ = 4.577, df = 1, *P* = 0.032) and maternal age at oviposition (Wald χ^2^ = 9.446, df = 1, *P* = 0.002). Specifically, offspring from high-density-reared and old mothers had lower predation incidence than those from low-density-reared and young mothers, respectively (Table 2). The interaction between maternal rearing density and age was nonsignificant (Wald χ^2^ = 1.632, df = 1, *P* = 0.201). In contrast, offspring latency to attack was neither affected by maternal rearing density (Wald χ^2^ = 0.537, df = 1, *P* = 0.464), nor maternal age at oviposition (Wald χ^2^ = 1.395, df = 1, *P* = 0.238), nor their interaction (Wald χ^2^ = 0.058, df = 1, *P* = 0.810) (Table 2).

**Table 2:** Predation incidence and latency to attack of *Amblyseius herbicolus* offspring whose mothers were reared under different densities and at different ages during oviposition.

Density	Age	*n*	Predation (%)	*n*	Latency (min)
**Low**	Young	35	57.1%^b^	20	110 (45 to 153.75)
	Old	35	40.0%^b^	14	60 (33.75 to 112.5)
**High**	Young	35	51.4%^b^	18	150 (60 to 165)
	Old	35	17.1%^a^	6	67.5 (48.75 to 131.25)

Each offspring was offered one *Carpoglyphus lactis* protonymph. Different letters (a, b) after percentage results denote significant differences from pairwise contrasts of estimated marginal means (*P* < 0.05). Attack latencies were expressed as medians with IQR in brackets.

### Experiment 3: superfluous killing

**Figure 6:**
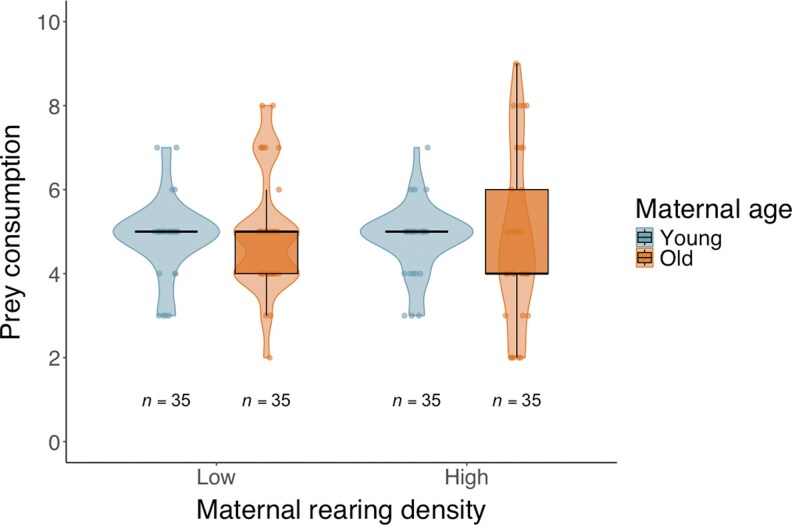
Prey consumption of *Amblyseius herbicolus* offspring whose mothers were reared under different densities and at different ages during oviposition. Each offspring was given 10 *Carpoglyphus lactis* larvae. Each violin shows the density of observations; dots represent individual consumptions; boxplots indicate median, IQR, and 1.5 × IQR whiskers. Sample sizes (*n*) are indicated below each box.

All killed *C. lactis* larvae were fully consumed, as indicated by the completely deflated prey corpses; partial consumption was never observed (Fig. 1f). Furthermore, the number of *C. lactis* larvae consumed was not significantly affected by maternal rearing density (GLMs: Wald χ^2^ = 0.073, df = 1, *P* = 0.787), nor maternal age at oviposition (Wald χ^2^ = 0.003, df = 1, *P* = 0.957), nor the interaction between the 2 factors (Wald χ^2^ = 0.023, df = 1, *P* = 0.879; Fig. 6).

## Discussion

Our study demonstrates that maternal age and rearing density both affect offspring foraging behaviors in *A. herbicolus*. Offspring of older mothers generally exhibited more conservative foraging strategies: reduced prey consumption and lowered predation incidence. High-density rearing of mothers increased offspring survival under restricted prey availability, but maternal age at oviposition did not influence offspring survival. Superfluous killing was not observed in larvae. This suggests that offspring of older and high-density mothers expressed conservative foraging phenotypes, rather than wasteful phenotypes in the offspring of younger and low-density mothers. Whether later life stages of *A. herbicolus* may display superfluous killing, particularly under high prey availability, remains to be tested. Finally, high maternal density enhanced survival and reduced prey consumption in offspring of young mothers only.

### Maternal provisioning and egg size plasticity

The observed increased survival (Table 1) and reduced predation incidence (Table 2) of offspring from high-density mothers were likely mediated by enhanced maternal provisioning, reflected in egg size. We found that mothers reared at high density produced larger eggs than those reared at low density, which is consistent with observations that maternal density can influence egg provisioning (Yanagi et al. 2013; Maeno et al. 2025). For example, desert locusts (*Schistocerca gregaria*) and seed beetles (*Callosobruchus chinensis*) produced larger eggs when mothers experienced crowding, and *Daphnia* showed a similar response at intermediate densities (Burns 1995; Yanagi et al. 2013; Maeno et al. 2025). Moreover, the hatchlings of desert locust mothers laid under crowed condition had higher tolerance to starvation than the smaller eggs lay by isolated mothers (Maeno et al. 2025). Nevertheless, the effects of maternal population density on egg size are not universal: the seed beetle (*Callosobruchus maculatus*) produced larger eggs at low density but smaller ones at high density (Fox and Savalli 1998), and no effect was observed in the soil mite *Sancassania berlesei* (Plaistow et al. 2007). These contrasting outcomes suggest that density effects are context-dependent and can often be adaptive when maternal environment reliably predicts offspring environment (Fox et al. 1997; Fox and Mousseau 1998; Badyaev and Uller 2009).

Contrary to earlier reports, we did not detect differences in egg size produced by young and old mothers (Zhang et al. 2024). This discrepancy could be due to rearing differences—here females were kept in groups of 2 or 5, rather than in isolation—or because the age separation between Young and Old oviposition females was too narrow to capture previously reported effects; further investigation is needed to clarify these possibilities.

### Maternal influence on offspring behavior

Offspring of older mothers displayed a more conservative predation phenotype: reduced prey consumption and lower predation incidence toward relatively large prey. Such behavioral tendencies may reflect underlying “personality traits,” which are now recognized as widespread in both vertebrates and invertebrates (Biro and Stamps 2008; Nguyen and Schausberger 2025). For instance, a survey suggests that personality traits such as boldness can be positively linked to food consumption, with “bold” individuals showing increased food intake (Biro and Stamps 2008).

Also maternally experienced population density affected offspring behavior: offspring of high-density mothers exhibited reduced predation incidence against unfamiliar prey. Parental environments are increasingly recognized as drivers of offspring behavioral strategies (Bitume et al. 2013, 2014). Variation in maternal provisioning may further induce differences in offspring responses (Plaistow et al. 2015). For instance, the asset protection principle (Clark 1994) suggests that offspring from high-density mothers, being larger at hatching (ie larger egg size), may represent more precious “assets” that behave more cautiously.

### Implications and future directions

Conservative foraging strategies were more common among offspring of older or high-density mothers, which may represent adaptive responses to anticipated resource scarcity or competition, and an adjusted asset protection strategy. This aligns with other findings that maternal environments can serve as predictive cues for offspring, which enables adaptive parental effects (Fox and Mousseau 1998; Badyaev and Uller 2009). For example, food-limited crustacean (*Daphnia magna*) mothers produced offspring with reduced feeding rates, which suggests a preadaptation for low-resource environments (Garbutt and Little 2014). Similar processes may operate in *A. herbicolus* that influences both individual fitness and population dynamics.

Whether these early-life foraging strategies persist across later life remains to be tested. Potential tradeoffs with other life-history traits, such as reproduction or dispersal, may emerge and should be investigated in future studies. Moreover, the mechanisms underlying these maternal effects remain elusive: it is unclear whether they stem from differential nutrient provisioning, epigenetic regulation, or other maternal pathways.

## Conclusion

Our results indicate that both age and rearing density of *A. herbicolus* mothers can induce variation in offspring prey consumption, survival, and predation incidence. These findings highlight that maternal effects are dynamic rather than static. Moreover, this study provides insights into the role of maternal effects in shaping predator–prey interactions and population dynamics.

## Data Availability

Analyses reported in this article can be reproduced using the data provided by Zhang et al. (2026).
